# The diagnostic accuracy of diabetes retinopathy screening by ophthalmic clinical officers, ophthalmic nurses and county ophthalmologists against a retina specialist in 2 selected county referral hospitals, Kenya

**DOI:** 10.3389/fopht.2023.1082205

**Published:** 2023-04-14

**Authors:** Jane Rahedi Ong’ang’o, Olga Mashedi, Micheal Gichangi, Richard Kiplimo, Joseph Nyamori, Kennedy Alwenya

**Affiliations:** ^1^ Centre for Respiratory Diseases, Kenya Medical Research Institute, Nairobi, Kenya; ^2^ Ophthalmic Services Unit, Ministry of Health, Nairobi, Kenya; ^3^ Department of Ophthalmology, University of Nairobi, Nairobi, Kenya; ^4^ Monitoring and Evaluation Unit, Fred Hollows Foundation Kenya, Nairobi, Kenya

**Keywords:** diabetes retinopathy, sensitivity, specificity, ophthalmic nurse, ophthalmic clinical officer, county ophthalmologist

## Abstract

**Background:**

Diabetes is rapidly becoming a major cause of blindness among Kenyans, with the prevalence of any form of diabetes retinopathy (DR) ranging from 36% to 41%. Globally DR leads as a cause of vision loss in working age adults. In Kenya, specialized examinations are only available at national and some county referral hospitals through retina specialists, ophthalmologists or trained technicians. Thus, low coverage of retinal assessment and inadequate access to this service. An innovative DR fundus camera screening service run by ophthalmic nurses (ONs), ophthalmic clinical officers (OCOs) and county ophthalmologists was established since 2018.

**Objectives:**

The purpose of this study was to investigate the diagnostic accuracy of DR digital retinal camera screening by ONs, OCOs and county ophthalmologist against that of a retina specialist measured by sensitivity and specificity as the primary outcomes.

**Methods:**

Cross sectional study conducted at 2 referral hospitals in Kenya. Using a Canon CR-2AF digital retinal camera patients with diabetes had a standard single shot of 45 degree view of the retina captured as image in each eye. This was graded for DR using the International Clinical Diabetic Retinopathy (ICDR) severity scale. All photos taken by the first graders (ON/OCO) were later assessed by the county hospital ophthalmologist who was blinded to their readings. The third grader (retina specialist) similarly was blinded to the readings of the first and second graders and assessed all the images from the 2 hospitals also using ICDR.

**Results:**

A total of 308 patients with diabetes (median age 58 IQR 56-60, 53% female) were enrolled in the study. Sensitivity to identify any DR was (81.3%, 80.6%, and 81.54% for the OCO, ON and county ophthalmologist respectively). The corresponding specificities were 92.7%, 92.8% and 92.59%. Analysis of diagnostic accuracy of non-sight threatening DR against sight threatening DR revealed lower sensitivity for the three cadre groups although specificity remained high.

**Conclusions:**

In this study, ON and OCO with basic training in DR screening and photo grading performed screening of DR with high specificity. However, the sensitivity to detect sight threatening DR was generally low by all the cadres which may leave severe forms of DR undetected.

## 1 Introduction

The risk of vision loss in people with diabetes is up to 25 times greater than in people who do not have diabetes (1). Approximately one-third of people with diabetes have diabetes-related eye disease, and the risk increases with the duration of diabetes, poor glycemic control, and the presence of hypertension (2). Diabetic retinopathy (DR) is the most common of the Diabetic eye diseases (DEDs) and it is currently the leading cause of vision loss in working age adults worldwide (3). DR is predicted to become one of the leading causes of blindness globally within the next 20 years (4). People with severe vision loss require additional health resources and endure reduced levels of physical, emotional, and social well-being (5, 6).

Non-communicable diseases (NCDs) such as diabetes are on the rise in Kenya. It is estimated that more than half a million people have diabetes in the country, and that almost half of these are undiagnosed (4). With the steady rise in diabetes, the prevalence of diabetes related complications such as DR is also increasing. With a nationwide prevalence of 2.2%, diabetes is rapidly becoming a leading cause of blindness in Kenya (4). The Kenya STEPwise survey for NCDs risk factors in 2015 reported that 40% of those known to have diabetes in Kenya were on treatment (7). The prevalence of any form of DR in Kenya has been reported from 36% to 41%, and 9% to 14% require laser eye therapy (8, 9).

It is estimated that approximately a third of people with diabetes have at least one form of DR at any time point, and approximately 10% of people with diabetes have sight-threatening DR that requires treatment (10, 11).

In Kenya, retinal assessments by specialist ophthalmologists or trained technicians are only available at national and county referral hospitals. This has resulted in low coverage of retinal assessment as there is inadequate access to this service. To address this shortage in Kenya, an initiative training of ophthalmic nurses (ONs) and ophthalmic clinical officers (OCOs) in Migori and Baringo county hospitals commenced in 2018 supported by the Fred Hollows foundation.

The main objective of this study was to investigate the diagnostic accuracy of DR screening by ONs, OCOs and county ophthalmologist against that of a retina specialist. The primary outcomes were sensitivity, specificity, positive and negative predictive values.

## 2 Methods and materials

### 2.1 Study design and sites

This was a cross sectional study that analyzed the accuracy screening of DR done by the OCOs, ONs and ophthalmologists at the diabetic medical out-patient clinics of the county referral hospitals in Migori and Baringo. These health facilities are established sites for DR screening in terms of diagnostic infrastructure including availability of computerized retinal (fundus) cameras, have regular functioning medical out-patient diabetic and eye clinics, and there is availability of trained nurses and clinical officers in ophthalmic care which includes fundus photography and intra vitreal ant- VEGF injections. In addition, each of these sites have a resident ophthalmologist who oversees eye care of the health facility.

### 2.2 Study inclusion and exclusion criteria

#### 2.2.1 Inclusion criteria for health care workers

The OCOs and ONs in the two county hospitals needed to have basic clinical medicine and nursing training, respectively, post-basic training in ophthalmology and DR screening. The 2 cadres had been trained on photo grading at the Kenyatta National Hospital (KNH) endocrinology center in January 2019 before the start of the study in October 2019. They were required to have provided eye care for at least 6 months at the county referral hospital. The ophthalmologist was a qualified medical doctor with post-graduate training in ophthalmology and DR screening.

#### 2.2.2 Inclusion criteria for diabetes patients to be examined

Diabetes patients aged ≥18 years attending the outpatient diabetic clinic who provided informed consent were eligible. Those who were too sick to undergo retinal photography/eye examination or whose ocular media was too hazy to allow retina photography were excluded from participating in the study.

### 2.3 Diabetic retinopathy screening

Consecutive patients attending diabetic medical clinic during the study period were recruited until the minimum sample size was achieved for each site. The DR screening was performed using desktop retinal cameras and the assessment done by the ONs and OCOs were assessed against the readings of a retina specialist who was considered as the reference standard. The first level screening was conducted by ONs and OCOs, while the second level screening was conducted by qualified doctor ophthalmologist working at the county referral hospital. The third level screening was performed by a national level retina specialist who assessed all the images from the two county hospitals.

### 2.4 Study procedures

Staff training in data collection and approval meetings with County Health Management were conducted in advance.

#### 2.4.1 Ophthalmic examination and grading of diabetic retinopathy

The diabetes patients underwent a complete ophthalmic examination, in which fundus imaging was performed and subsequently graded for DR. In addition to DR screening diabetic macular edema (DME) screening was performed using the same color fundus images and was classified as apparently present or apparently absent for each image. This study was limited to hard exudates to determine presence of DME because of the non-stereoscopic fundus photos. Using the Clinical Practice Guidelines for Diabetic Retinopathy in Kenya (12), no DME was defined as no exudates within macula vascular arcades on fundus photo and DME was hard exudates within macular vascular arcades of fundus photo. We did not perform optical coherence tomography (OCT) for DME thus there was no study data on fovea involvement.

The ON and OCO took retinal photographs of each eye using a desktop fundus camera (Canon Digital Retinal Camera CR-2 AF). The images graded were all non-mydriatic standard field macula-centered single-shot photos. All patients were un-dilated and a standard single shot of 45 degree view of the retina was used to capture the image. The International Clinical Diabetic Retinopathy (ICDR) severity scale (13) was used to stage DR. The gradings were labelled R0 (No apparent diabetic retinopathy), R1 (Mild non-proliferative diabetic retinopathy), R2 (Moderate non-proliferative diabetic retinopathy), R3 (Severe non-proliferative) and R4 (Proliferative retinopathy).

All photos taken by the first grader (ON and OCO) were later assessed by the county hospital ophthalmologist to grade the retina changes using the ICDR criteria. The second grader (county ophthalmologist) was blinded to the reading of the first grader. The third grader (retina specialist) similarly was blinded to the readings of the first and second graders and assessed all the images from the 2 hospitals using ICDR criteria. Patients with other eye diseases/ocular complications of diabetes were referred to the site ophthalmologist for further examination and treatment. This included patients with ungradable study images. For this study sight-threatening DR was defined as severe non-proliferative retinopathy or worse (R3, R4).

### 2.5 Sample size calculation and sampling

Sample size calculations were computed using Power Analysis and Sample Size Software (PASS NCSS) and guided with main reference of Obuchowski N and McClish D (14). The prevalence of sight-threatening DR among people with diabetes in Kenya was estimated to be 13.4% (15). The PASS calculated a total sample size that achieves 90% power to detect a change in sensitivity from 0.999 to 0.912 using a two-sided binomial test and 31% power to detect a change in specificity from 0.999 to 0.993 using a two-sided binomial test. The target significance level was 0.05. To achieve the required sensitivity and specificity, the minimum sample size of diabetes patients was determined to be 296 to be distributed in the 2 study sites proportionally. Using the previous year annual caseload of diabetes patients that attended the medical clinics of each of these sites, which was 960 in Migori and 700 in Baringo county hospitals, the sample size was proportionately distributed based on the diabetic patient case load of the sites (Migori 170 and Baringo 126). At the sites, the ONs and the OCOs had equal numbers to grade for DR.

### 2.6 Data management and analysis

Computer tablets installed with electronic data collection tool were used. The data collection process used electronic databases which allowed offline retrieval and online uploading for transmission to the central database. The design of the databases ensured integrity and security of stored data and export of data for analysis. Each retinal photo of the diabetic patient with its unique identifier was stored securely within a folder on a desktop computer and in an external hard drive as a back-up. Each study OCO, ON, and ophthalmologist had a unique study identifier against their records and images. A blinded retina specialist at a remote site graded all the retina images using a customized electronic platform designed for this study.

The primary outcomes were the sensitivity, specificity, positive and negative predictive value of the DR grading performed by the ON, OCO, the county ophthalmologist versus the retina specialist’s grading. The sensitivity and specificity analysis was performed in 2 ways; no DR against any DR and non-sight threatening DR against sight threatening DR. The diagnostic accuracy analysis was derived after exclusion of ungradable images and considering the eye as the unit of analysis. Statistical analysis was performed using standard software package (Stata, version 14.0; Stata Corp)

### 2.7 Ethical considerations

This study received KEMRI Scientific and Ethic Review Unit (SERU) approval (SERU 3856) before implementation. Written informed consent was obtained from all participants (or legally acceptable representative) before any study-related procedures were performed. Each informed consent form included the elements required by the international Good Clinical Practice and adhered to the ethical principles that have their origin in the Declaration of Helsinki.

## 3 Results

Data were collected from July to December 2019. A total of 308 patients with diabetes were enrolled into the study with 127 at Baringo County Referral hospital and the remaining 181 at Migori County Referral hospital. The median age was 58 (IQR 56-60; 84% aged ‗46 years) with 53% females.

### 3.1 Diabetic retinopathy grading

**Figure 1:**
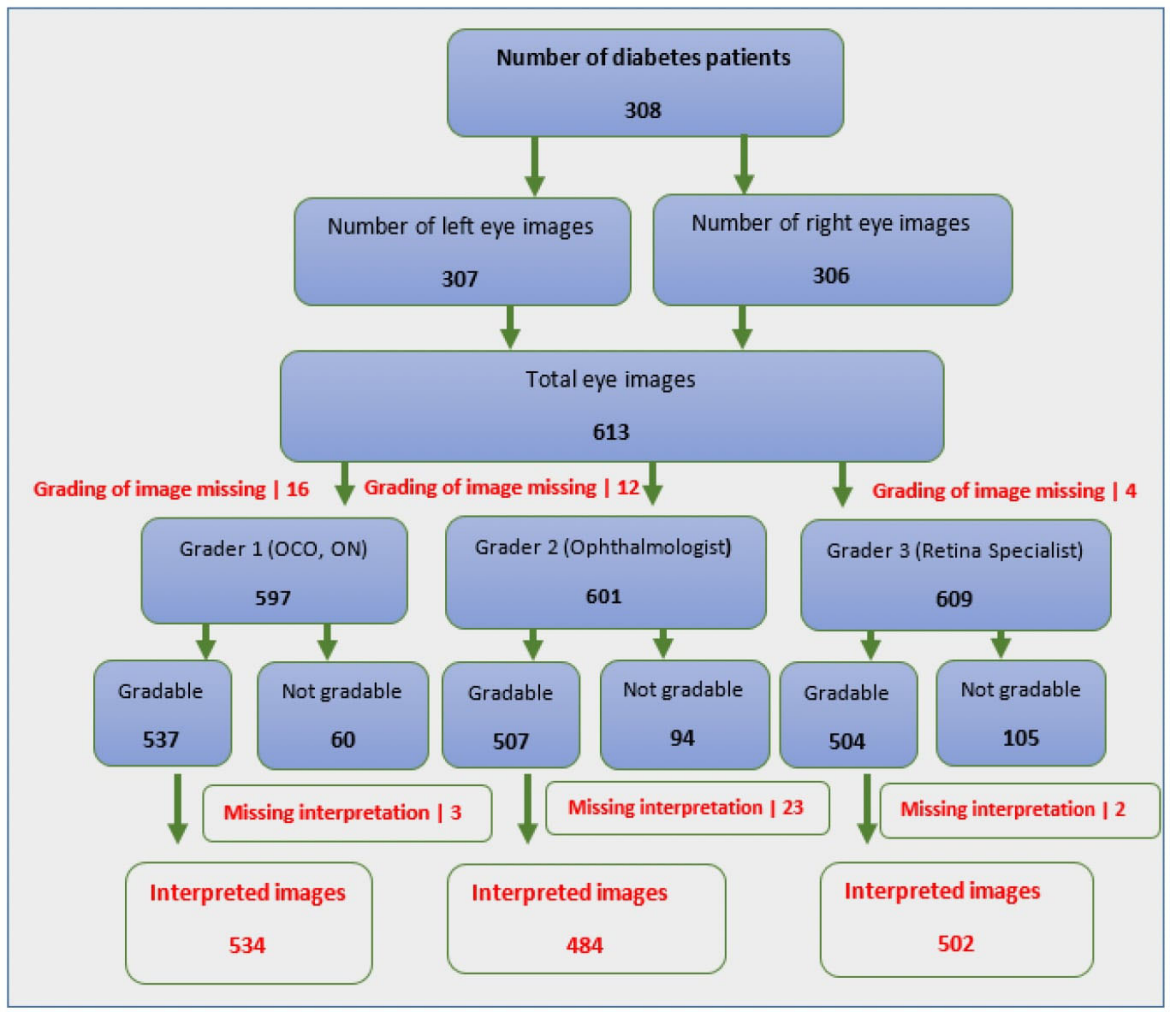
Schematic diagram of eye images graded and interpreted by graders 1, 2 and 3.

A total of 597 eye images were examined by first graders for grading of diabetic retinopathy, of these 534 (89.4%) were gradable while the second graders examined a total of 601 eye images of which 484 (80.5%) were gradable and the third grader examined 609 eye images and was able to grade 504 (82.8%) (**Figure 1**)

**Figure 2:**
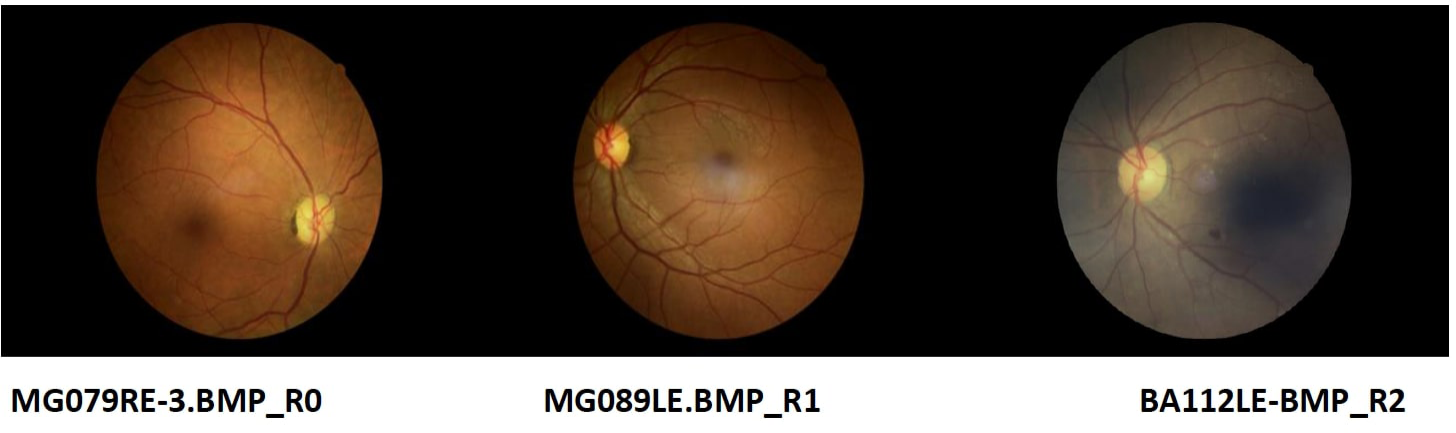
Examples of retina images graded.

Most of the grading of the eye images were categorized as having no DR by all the 3 levels of graders as follows 1^st^ 71.7% CI (67.73%, 75.39%), 2^nd^ 69.83% CI (65.58%, 73.77%) and 3^rd^ 69.52% CI (65.34%, 73.41%) as indicated in **Table 1**. This indicated the prevalence of DR as 28.28% CI (24.61%, 32.26%) for 1^st^ graders, 30.17% CI (26.23%, 34.42%) for 2^nd^ graders and 30.48% CI (26.60%, 34.66%) for 3^rd^ grader. **Figure 2** shows some of the images graded. The images MG079RE-3.BMP_ RO, MG089LE.BMP_R1. BA112LE-BMP R2 were graded as R0, R1 and R2 respectively by grader 3. Grading of the same images by grader 1 was R0, RO, and R2, while grader 2 categorized these as R0, R0 and R2 in the same respective manner.

**Table 1:** Retinopathy grading of 1^st^, 2^nd^ and 3^rd^ graders.

Retinopathy grading	1^st^ grader		2^nd^ grader		3^rd^ grader	
	n	95% (CI)	n	95% (CI)	n	95% (CI)
R0=no DR	383	71.72% (67.73%, 75.39%)	338	69.83% (65.58%, 73.77%)	349	69.52% (65.33%, 73.4%)
R1=Mild background DR	83	15.54% (12.7%, 18.88%)	84	17.35% (14.22%, 21%)	90	17.92% (14.8%, 21.54%)
R2= Moderate DR	27	5.05% (3.48%, 7.28%)	28	5.78% (4.01%, 8.26%)	16	3.18% (1.95%, 5.14%)
R3=Severe non proliferative	23	4.3% (2.87%, 6.4%)	22	4.54% (3%, 6.81%)	26	5.17% (3.54%, 7.5%)
R4= Proliferative retinopathy	18	3.37% (2.13%, 5.29%)	12	2.47% (1.41%, 4.32%)	21	4.18% (2.73%, 6.33%)
Total	534	100%	484	100%	502	100%

Grader 1, ON or OCO; Grader 2, County Ophthalmologist; Grader 3, Retina Specialist.

Macular assessment by the 3 graders categorized most images as no DME with 81.3%, 80.6% and 79.68% as reported by graders 1,2 and 3 respectively (**Table 2**).

**Table 2:** Diabetes macula oedema staging by the 3 levels of grading.

	Grader 1	Grader 2	Grader 3
Maculopathy grading	n (%)	n (%)	n (%)
M0 (No DME)	434 (81.3%)	390 (80.6%)	400 (79.68%)
M1 (DME)	82 (15.4%)	82 (16.9%)	90 (17.9%)
Ungradeable	18 (3.4%)	12 (2.5%)	12 (2.39%)
Total Images	534	484	502

### 3.2 Overall sensitivity, specificity, positive predictive value and negative predictive value

Using the no DR vs any DR classification, the overall sensitivities of the ON, OCO (1^st^ grader) and county ophthalmologist (2^nd^ grader) on rating DR against the retina specialist were 80.9% CI (73.8, 86.8) and 81.5% CI (77.97,85.11), respectively. The specificities on the same among 1^st^ and 2^nd^ graders were 92.8% CI (89.5, 95.3) and 92.59 CI (90.18, 95.00). The positive predictive values (PPVs) were 83.1% CI (76.1, 88.8) and 81.54% CI (77.97, 85.11) for 1^st^ and 2^nd^ graders respectively while their negative predictive values (NPVs) were 91.7% CI (88.3, 94.4) and 92.59% CI (90.18, 95.00) (**Table 3**).

**Table 3:** Overall Sensitivity, Specificity, Positive predictive value and Negative predictive value.

Characteristic		(No DR vs Any DR)				(non-sight threatening DR vs sight threatening DR)			
		Grader 1 vs Grader 3		Grader 2 vs Grader 3		Grader 1 vs Grader 3		Grader 2 vs Grader 3	
		PE %	(95% CI)	PE %	(95% CI)	PE %	(95% CI)	PE %	(95% CI)
Overall	Sensitivity	80.9 (123/153)	(73.8, 86.8)	81.54 (106/130)	(77.97, 85.11)	52.2 (24/46)	(36.9, 67.1)	52.5 (21/40)	(36.1, 68.5)
	Specificity	92.8 (321/346)	(89.5, 95.3)	92.59 (300/324)	(90.18, 95.00)	96.8 (425/439)	(94.7, 98.2)	97.6 (404/414)	(95.6, 98.8)
	Positive predictive value	83.1 (123/148)	(76.1, 88.8)	81.54 ((106/130)	(77.97, 85.11)	63.2 (24/38	(46, 78.2)	67.7 (21/31)	(48.6, 83.3)
	Negative predictive value	91.7 (321/350)	(88.3, 94.4)	92.59 (300/324)	(90.18, 95.00	95.1 (425/447)	(92.6, 96.9)	95.5 (404/423)	(93.1, 97.3)

PE, Point estimate; Grader 1, ON or OCO; Grader 2, County Ophthalmologist; Grader 3, Retina Specialist.

The overall sensitivities of the other analysis based on non-sight threatening vs sight threatening were 52.2% CI (36.9, 67.1) and 52.5% CI (36.1,68.5), for 1^st^ grader and 2^nd^ grader respectively. The specificities between the 1^st^ and 2^nd^ graders on the same were 96.8% CI (94.7, 98.2) and 97.6% CI (95.6, 98.8). Overall PPVs were 63.2% CI (46, 78.2) and 67.7% CI (48.6, 83.3) respectively for 1^st^ and 2^nd^ graders while the NPVs were 95.1% CI (92.6, 96.9) and 95.5% (93.1, 97.3) (**Table 3**).

### 3.3 Sensitivity and specificity analyzed against level of grader and by county

The 1^st^ and 2^nd^ graders of Baringo county hospital performed better than the Migori county graders in classifying no DR against any form of DR with sensitivities of 95% CI (86.1.99) and 94.3% CI (91,97.8) respectively compared to 71,7% CI (61.4,80.6) and 72.6% CI (67.42, 78.07) for the same graders in Migori. Generally, the sensitivity to identify non-sight threatening DR vs sight threatening DR was low in both counties with both 1^st^ and 2^nd^ Migori county hospital graders having better sensitivity levels of 76.2% CI (71.39, 80.99) and 63.2% CI (57.4, 68.91) respectively compared to Baringo county with 32% CI (25.22, 38.78) and 42.9% CI (35.71, 50.01) sensitivities of graders 1 and 2 (**Table 4**).

**Table 4:** Sensitivity and Specificity segregated by grader level and County.

Characteristic		(No DR vs Any DR)				(non-sight threatening DR vs sight threatening DR)			
		Grader 1 vs Grader 3		Grader 2 vs Grader 3		Grader 1 vs Grader 3		Grader 2 vs Grader 3	
		PE %	(95% CI)	PE %	(95% CI)	PE %	(95% CI)	PE %	(95% CI)
Baringo CRH	Sensitivity	95 (57/60)	(86.1, 99)	94.3 (50/53)	(91, 97.68)	32 (8/25)	(25.22, 38.78)	42.9 (9/21)	(35.71, 50.01)
	Specificity	88 (117/133)	(81.2, 93)	87.8 (115/131)	(83.05, 92.52)	99.4 (156/157)	(98.21, 100.52)	98.8 (161/163)	(97.18, 100.36)
	Positive predictive value	78.1 (57/73)	(66.9, 86.9)	75.8 (50/66)	(69.57, 81.95)	88.9 (8/9)	(84.32, 93.45)	81.8 (9/11)	(76.25, 87.39)
	Negative predictive value	97.5 (117/120)	(92.9, 99.5)	97.5 (115/118)	(95.18, 99.73)	90.2 (156/173)	(85.85, 94.5)	93.1 (161/173)	(89.39, 96.73)
Migori CRH	Sensitivity	71.7 (66/92)	(61.4, 80.6)	72.7 (56/77)	(67.42, 78.04)	76.2 (16/21)	(71.39, 80.99)	63.2 (12/19)	(57.4, 68.91)
	Specificity	95.8 (204/213)	(92.1, 98)	95.9 (185/193)	(93.48, 98.23)	95.4 (269/282)	(93.03, 97.75)	96.8 (243/251)	(94.72, 98.91)
	Positive predictive value	88 (66/75)	(78.4, 94.4)	87.5 (56/64)	(83.56, 91.44)	55.2 (16/29)	(49.57, 60.77)	60 (12/20)	(54.16, 65.84)
	Negative predictive value	88.7 (204/230)	(83.9, 92.5)	89.8 (185/193)	(86.2, 93.41)	98.2 (269/274)	(96.67, 99.68)	97.2 (243/250)	(95.23, 99.17)

PE, Point estimate; Grader 1, ON or OCO; Grader 2, County Ophthalmologist; Grader 3, Retina Specialist OCO, Ophthalmic Clinical Officer; ON, Ophthalmic Nurse.

### 3.4 Image grading

Out of the 105 images captured as ungradable by the 3^rd^ grader, 28.5% (30/105) were captured as gradable by both 1^st^ and 2^nd^ graders. The 1^st.^grader did capture 43.8% (46/105) of them as gradable while 2^nd^ grader had 32.3% (34/105) as gradable (**Table 5**). On staging these images for DR, the 1^st^ grader interpreted 74% (34/46) as no DR, while 2^nd^ grader had 35% (12/34) as no DR (**Table 6**).

**Table 5:** The grading of the ungradable images of grader 3 by graders 1 and 2.

Level 1 grader	Level 2 grader			
	Gradable	Not gradable	Missing	Total
Gradable	30 (28.5%)	14 (13.3%)	2 (1.9%)	46 (43.8%)
Not gradable	4 (3.8%)	48 (45.7%)	2 (1.9%)	54 (51.4%)
Missing	0 (0%)	5 (4.7%)	0 (0%)	5 (4.7%)
Total	34 (32.3%)	67 (63.8%)	4 (3.8%)	105

**Table 6:** DR staging of the ungradable 3^rd^ grader images by 1^st^ and 2^nd^ graders.

Staging of DR	Grader 1 n (%)	Grader 2 n (%)
R0	34 (74%)	12 (35%)
R1	6 (13%)	8 (24%)
R2	2(4%)	4 (12%)
R3	3(7%)	1 (3%)
R4	0 (0%)	1 (3%)
Missing	1(2%)	8(24%)
Total	46 (100%)	34 (100%)

### 3.5 Macula oedema staging

Based on the interpretations of grader 3 as concerns diabetic macular oedema, (17.9%) 90/502 images were classified as DME. The DR staging of these images was as follows 12 (13.3%), 38 (42.2%), 11 (12.2%), 22 (24.5%) and 7 (7.8%) respectively for R0, R1, R2, R3 and R4 DR by the same grader (**Table 7**).

**Table 7:** Diabetes retinopathy staging against macula oedema staging by grader 3.

DR staging by grader 3	Macula Oedema staging by grader 3			
	M0	M1	Ungradable	Total
R0	333 (83.25%)	12 (13.3%)	4 (33.3%)	349 (69.5%)
R1	48 (12%)	38 (42.2%)	4 (33.3%)	90 (17.9%)
R2	5 (1.25%)	11 (12.2%)	0 (0%)	16 (3.2%)
R3	3 (0.75%)	22 (24.5%)	1 (8.4%)	26 (5.2%)
R4	11 (2.75%)	7 (7.8%)	3 (25%)	21(4.2%)
Total	400 (100%)	90 (100%)	12 (100%)	502 (100%)

## 4 Discussion

This study is one of the few studies that has evaluated the accuracy of DR screening by mid-level ophthalmic workers trained in ophthalmic assessments of DR in Kenya. This is important because evidence from low and middle income countries, regarding diagnostic accuracy of DR screening interventions, is known to be scarce.

In Kenya diabetic retinopathy is often diagnosed late, due to lack of access to screening services among other reasons. The number of ophthalmologists per population is small (for example in Sub-Saharan Africa it is estimated at 3.7 per million population) (16), and hence it is not feasible for ophthalmologists to provide screening services to all patients who need it. If other eye care workers can screen with reasonable accuracy, this can increase access to screening services and prevent blindness from DR in the population.

The prevalence of any form of DR detected by the different levels of graders was comparable at 28.28% (1^st^ graders), 30.17% (2^nd^ graders) and 30.48% (3^rd^ grader). These prevalence rates are consistently associated with older age as reported by van Leiden HA et al. (17). A majority (84%) of our study participants were more than 45 years.

The overall sensitivity and specificity of the ON, OCO and the county ophthalmologist to detect any form of DR with reference to the retina specialist was 80.9% (95% CI; 73.8, 86.8) and 81.54% (95% CI; 77.97,85.11) respectively. These sensitivity levels are of lower level in comparison with a systematic review conducted by Piyasena et al. which reported a pooled sensitivity of detection of any level of DR as 86% (95% CI 85,87) and a pooled specificity value of 91% (95% CI 90,92) (18). The study results indicate the need to improve the proficiency of the trained health care workers for more reliable DR detection staging.

The grading of any form of DR by the ON and the OCO against the retina specialist was comparable with sensitivities of 80.6% and 81.3% respectively. Considering that these 2 cadres have different basic medical training, this finding reassures us of their competence to perform the screening independently in primary care. With the current era of artificial intelligence (AI) the machine learning process is going to help in the same way, i.e., helping the people in the community to detect the eyes with DR and those who need to be referred to a retina specialist (19, 20). However, till we get a wider availability of AI systems globally, the role of the ophthalmic nurses and ophthalmic clinical officers is very crucial in the screening of eyes with DR

The better performance of Baringo County in reporting any form of DR may have been contributed by the county ophthalmologist who had more than 90% sensitivity. This good accuracy could be attributable to the specialist eye medical training and the work experience of this expert, as has been reported by Bragge P et al. (21). Using sight-threatening DR classification the overall sensitivity and specificity was much lower at all levels of the graders at the 2 hospitals. Despite this observation there seemed to be a better performance from the Migori study site compared to Baringo on categorizing sight threatening DR. A probable explanation to this may be that the Migori site had the advantage of having been exposed more to incident cases of sight threatening DR than Baringo because its DR screening program was initiated 6 months earlier than the other site. A factor which supports more experience contributing to better performance. Unfortunately, the study did not investigate individual factors of the graders which may have contributed to these differences. The low overall sensitivity for detecting sight threatening DR is below the recommended values for DR screening programs of over 80% sensitivity but within the 95% specificity (22). This may imply that there are severe forms of DR which might not be detected by both 1^st^ and 2^nd^ graders an indication of a possible risk gap of diabetes patients progressing into blindness. A more accurate DR screening program would minimize this.

This study found a wide discrepancy in the grading of the 3^rd^ grader’s ungradable images between both the 1^st^ and 2^nd^ graders and the 3^rd^ grader. Most of the images that the 1^st^ and 2^nd^ grader deemed gradable were labeled as having no DR by these graders. This finding calls for the re-training of these graders on gradability of images, emphasizing the need of precisely recognizing the ungradable images and as a result, being referred for additional assessment by a retina specialist or ophthalmologist. By doing this, the possibility of missing pathology that could jeopardize vision would be reduced.

About 87% of images categorized as DME by grader 3 also had some form of DR at different stages and about 33% of these macula oedema images were categorized as sight threatening DR. These findings support the importance of performing both DR grading and screening for macular edema as the latter may be indicative of DR (23). The 13% macula oedema images that did not have any form of DR may be representing the DME patients who often self- present earlier to clinics because of macula reversible visual impairment which if treated with anti-vascular endothelial growth factor (antiVEGF) injections retards progression of DR to neovascularization.

Potential limitation of our study was use of one photographic field protocol and not the gold standard of 7 field ETDRS photos to capture DR lesion. The possibility of missing some of the peripheral retinal lesions and underestimating DR grading has been reported by Srihatrai P et al, who found that 5-field photography was more sensitive than single-field photography for DR detection (24). Another limitation was that the study did not describe the ungradable images to define cause. Probably as has been reported that the most common cause of ungradable images is cataract (25). This may apply to this situation considering that cataract is common in Kenya especially in adults aged 50 years and older (26). Also, the fact that the study used non-mydriatic cameras, this may have contributed to the images being ungradable. This study focused on hospitals that had manpower and machines that could perform DR screening, thus the prevalence of DR reported in this study may not be representative of various health facilities that receive diabetes patients. This is because the population sample from patients diagnosed with diabetes at lower level health facilities like dispensaries may be different compared to that at the county referral hospital.

The use of non-mydriatic imaging in this study was a strength that enabled reduced screening time allowing more diabetes patients to receive service over a shorter time than would be. In addition, this allowed less inconvenience for patients and the taking of study images by the mid-level ophthalmic workers was easy.

Conclusions and Recommendations

The ONs and the OCOs may be used reliably for screening any form of DR as 1^st^ graders. This may contribute to improved patient access and overall assessment rates of DR screening at primary care level. To improve the proficiency skills of detecting specific stages of DR, all cadres including the ophthalmologists should be re-trained for specific staging of DR. In addition, after training they will require continuous assessment to ensure their proficiency skills and accreditation as ophthalmic workers are maintained.

Our study did not evaluate the quality control system in place for the DR screening program in the two study sites. There is need to assess its adequacy to support the performance of DR screening programs.

## Data availability statement

The original contributions presented in the study are included in the article/supplementary material. Further inquiries can be directed to the corresponding author.

## Ethics statement

The studies involving human participants were reviewed and approved by Scientific Ethics and Review Unit, Kenya Medical Research Institute. The patients/participants provided their written informed consent to participate in this study.

## Author contributions

All authors contributed to the conception and design of the study. RK organized the data base, JO and RK did the analysis. JO wrote the first draft of the manuscript. JO, OM, RK, MG, JN and KA contributed to manuscript revisions, read, and approved the submitted version. All authors contributed to the article and approved the submitted version. 

## References

[1] Williams R Airey M Baxter H Forrester J Kennedy-Martin T. Girach a epidemiology of diabetic retinopathy and macula oedema: A systematic review. Eye (2004) 18:963–83. doi: 10.1038/sj.eye.6701476 15232600

[2] Yau JWY Rogers SL Kawasaki R Lamoureux EL Kowalski JW Bek T. Global prevalence and major risk factors for diabetic retinopathy. Diabetes Care (2012) 35(3):556–64. doi: 10.2337/dc11-1909 PMC3322721 22301125

[3] Resnikoff S Pascolini D Etya’ale D Kocur I Pararajasegaram R Pokharel GP. Global data on visual impairment in the year 2002. Bull World Health Organ (2004) 82:844–51. PMC2623053 15640920

[4] Atlast D. IDF diabetes atlas. International diabetes federation. 9th ed. (2019). Retrieved from: http://www.idf.org/about-diabetes/facts-figures.

[5] Coyne KS Margolis MK Kennedy-Martin T Baker TM Klein R Paul MD. The impact of diabetic retinopathy: Perspectives from patient support groups. Family Pract (2004) 21(4):447–53. doi: 10.1093/fampra/cmh417 15249536

[6] Huang ES Brown SES Ewigman BG Foley EC Meltzer DO. Patient perceptions of quality of life with diabetes-related complications and treatments. Diabetes Care (2007) 30:10. doi: 10.2337/dc07-0499 PMC2288662 17623824

[7] Ministry of Health Kenya. Kenya STEPwise survey for non-communicable diseases risk factors 2015 report. Nairobi: GOK (2015).

[8] Njambi L. Prevalence of diabetic retinopathy and barriers to uptake of diabetic retinopathy screening at embu provincial general hospital, central Kenya. East Afr J Ophthalmol (2012) 16(1).

[9] Mathenge W Bastawrous A Peto T Leung I Yorston D Foster A. Prevalence and corelates of diabetic retinopathy in a population-based survey of older people in nakuru, Kenya. Ophthalmic Epidemiologu (2014) 21(3):169–77. doi: 10.3109/09286586.2014.903982 24758280

[10] Yau JW Rogers SL Kawasaki R Lamoureux EL Kowalski JW Bek T. Global prevalence and major risk factors of diabetic retinopathy. Diabetes Care (2012) 35(3):556–64. doi: 10.2337/dc11-1909 PMC3322721 22301125

[11] Lee R Wong TY Sabanayagam C. Epidemiology of diabetic retinopathy, diabetic macular edema and related vision loss. Eye Vision (2015) 2(1):17. doi: 10.1186/s40662-015-0026-2 26605370 PMC4657234

[12] Ministry of Health. guidelines for the screening and management of diabetic retinopathy in Kenya (2017). Nairobi, Kenya.

[13] Wilkinson CP Ferris FL 3rd Klein RE Lee PP Agardh CD Davis M. Global diabetic retinopathy project group. proposed international clinical diabetic retinopathy and diabetic macular edema disease severity scales. Ophthalmology (2003) 110(9):1677–82. doi: 10.1016/S0161-6420(03)00475-5 13129861

[14] Obuchowski N McClish D. Sample size determination for diagnostic accuracy studies involving binormal ROC curve indices. Stat Med (1997) 16:1529–42. doi: 10.1002/(SICI)1097-0258(19970715)16:13<1529::AID-SIM565>3.0.CO;2-H 9249923

[15] Mathenge W Bastawrous A Peto T Leung I Foster A Kuper H. Prevalence of age-related macular degeneration in nakuru, Kenya: A cross-sectional population-based study. PloS Med (2013) 10(2):e1001393. doi: 10.1371/journal.pmed.1001393 23431274 PMC3576379

[16] Resnikoff S Lansingh VC Washburn L Felch W Gauthier TM Taylor HR. Estimated number of ophthalmologists worldwide (International council of ophthalmology update): Will we meet the needs? Br J Ophthalmol (2020) 104(4):588–92. doi: 10.1136/bjophthalmol-2019-314336 PMC7147181 31266774

[17] van Leiden HA Dekker JM Moll AC Nijpels G Heine RJ Bouter LM. Blood pressure, lipids, and obesity are associated with retinopathy: the hoorn study. Diabetes Care (2002) 25(8):1320. doi: 10.2337/diacare.25.8.1320 12145228

[18] Piyasena MMPN Murthy GVS Yip JLY Gilbert C Peto T Gordon I. Correction to: Systematic review and meta-analysis of diagnostic accuracy of detection of any level of diabetic retinopathy using digital retinal imaging. Syst Rev (2019) 8:106. doi: 10.1186/s13643-019-1023-7 31039817 PMC6492422

[19] Ferro Desideri L Rutigliani C Corazza P Nastasi A Roda M Nicolo M. The upcoming role of artificial intelligence (AI) for retinal and glaucomatous diseases. J Optometry (2022) 15(S1):S50–7. doi: 10.1016/j.optom.2022.08.001 PMC9732476 36216736

[20] Bellemo V Lim G Rim TH Tan GSW Cheung CY Sadda S. Artificial intelligence screening for diabetic retinopathy: The real-world emerging application. Curr Diabetes Rep (2019) 19(9):72. doi: 10.1007/s11892-019-1189-3 31367962

[21] Bragge P Gruen RL Chau M Forbes A Taylor HR. Screening for presence or absence of diabetic retinopathy: A meta-analysis. Arch Ophthalmol (2011) 129(4):435–44. doi: 10.1001/archophthalmol.2010.319 21149748

[22] Mead A Burnett S Davey C. Diabetic retinal screening in the UK. J R Soc Med (2001) 94(3):127–9. doi: 10.1177/014107680109400307 PMC1297928 11285793

[23] Riordan-Eva P. Eye. in current medical diagnosis and treatment. 42nd ed. Tierney LM McPhee SJ Papadakis MA, editors. New York: Lange Medical Books/McGraw-Hill (2003) p. 146–77.

[24] Srihatrai P Hlowchitsieng T. The diagnostic accuracy of single- and five-field fundus photography in diabetic retinopathy screening by primary care physicians. Indian J Ophthalmol (2018) 66(1):94–7. doi: 10.4103/ijo.IJO_657_17 PMC5778592 29283131

[25] Scanlon PH Foy C Malhotra R Aldington SJ. The influence of age, duration of diabetes, cataract, and pupil size on image quality in digital photographic retinal screening. Diabetes Care (2005) 28(10):2448–53. doi: 10.2337/diacare.28.10.2448 16186278

[26] Bastawrous A Mathenge W Nkurikiye J Wing K Rono H Gichangi M. Incidence of visually impairing cataracts among older adults in Kenya. JAMA Netw Open (2019) 2(6):e196354. doi: 10.1001/jamanetworkopen.2019.6354 31251374 PMC6604086

